# MFSD2B is a sphingosine 1-phosphate transporter in erythroid cells

**DOI:** 10.1038/s41598-018-23300-x

**Published:** 2018-03-21

**Authors:** Naoki Kobayashi, Shoko Kawasaki-Nishi, Masato Otsuka, Yu Hisano, Akihito Yamaguchi, Tsuyoshi Nishi

**Affiliations:** 1 Department of Biochemistry, Faculty of Pharmaceutical Sciences, Setsunan University, Hirakata, Osaka, 573–0101 Japan; 2 Faculty of Pharmaceutical Science, Teikyo Heisei University, Nakano-ku, Tokyo, 164–8530 Japan; 3 Department of Biomolecular Science and Regulation, Osaka University, Ibaraki, Osaka, 567–0047 Japan; 4 Department of Cell Membrane Structural Biology, Institute of Scientific and Industrial Research, Osaka University, Ibaraki, Osaka, 567–0047 Japan; 5 Faculty of Pharmaceutical Science, Osaka University, Suita, Osaka, 565–0871 Japan

## Abstract

Sphingosine 1-phosphate (S1P) is an intercellular signaling molecule present in blood. Erythrocytes have a central role in maintaining the S1P concentration in the blood stream. We previously demonstrated that S1P is exported from erythrocytes by a glyburide-sensitive S1P transporter. However, the gene encoding the S1P transporter in erythrocytes is unknown. In this study, we found that the mouse erythroid cell line, MEDEP-E14, has S1P export activity and exhibits properties that are consistent with those of erythrocytes. Using microarray analysis of MEDEP-E14 cells and its parental cell line, E14TG2a, we identified several candidate genes for S1P export activity. Of those genes, only one gene, *Mfsd2b*, showed S1P transport activity. The properties of S1P release by MFSD2B were similar to those in erythrocytes. Moreover, knockout of MFSD2B in MEDEP-E14 cells decreased S1P export from the cells. These results strongly suggest that MFSD2B is a novel S1P transporter in erythroid cells.

## Introduction

Sphingosine 1-phosphate (S1P) is a lipid mediator that is important for various cellular functions, such as in immunity^1,2^, vascularization^3^, and bone homeostasis^4^. S1P binds to its specific receptors (S1PR1-5) on the surface of target cells and causes specific cellular responses^5^. In mammals, S1P is abundant in the blood^6^ and plays an essential role in blood barrier function and lymphocyte egress from the thymus and secondary lymphoid organs into the blood stream^2^. Erythrocytes and endothelial cells constitutively release S1P to blood plasma and act as major contributors of plasma S1P.

We identified SPNS2 as an S1P transporter in endothelial cells and then demonstrated an essential role for SPNS2 in the migration of lymphocytes from the thymus and secondary lymphoid organs into the blood^7,8^. However, in SPNS2 knockout mice, S1P release from erythrocytes was normal, and the S1P concentration in blood plasma was maintained at approximately 60% of that in wild-type mice, indicating that SPNS2 is not the S1P transporter in erythrocytes^7^. These observations are consistent with a previous report indicating that the S1P concentration in blood plasma is maintained by S1P that is supplied by both endothelial cells and erythrocytes^9^.

S1P is synthesized from sphingosine by sphingosine kinase in erythrocytes^10,11^. Extracellular sphingosine is delivered to the inside of erythrocytes and converted to S1P by phosphorylation^10^. Intracellular S1P is released from erythrocytes in a time-dependent manner without any extracellular stimuli^10,12^. Using inside-out vesicles prepared from erythrocyte membranes, we demonstrated that S1P transport in erythrocytes requires ATP but not ATP hydrolysis and is suppressed by glyburide and vanadate^12^. These results strongly support the existence of S1P transporter molecules in erythrocytes. However, the gene for the erythrocyte S1P transporter has not yet been discovered.

In this study, we found that the mouse erythroid cell line MEDEP-E14 has S1P export activity that is similar to that in erythrocytes. Moreover, we identified a novel S1P transporter molecule in erythroid cells by using microarray analysis and a S1P transport assay.

## Results

### S1P is exported from the MEDEP-E14 erythroid cell line

Previously, we clearly demonstrated that S1P transport activity was detected in CHO cells that were overexpressing mouse sphingosine kinase 1 (CHO/SPHK1 cells) when an S1P transporter gene was expressed in the cells^13^. Thus, to identify the erythrocyte S1P transporter molecule, we cloned cDNAs of almost all transporters reported to be expressed in erythrocytes and investigated the S1P transport activity in the CHO/SPHK1 cells transfected with each transporter cDNA. However, none of the 23 transporter genes we cloned had S1P export activity in the cells (Supplemental Table 1).

**Figure 1:**
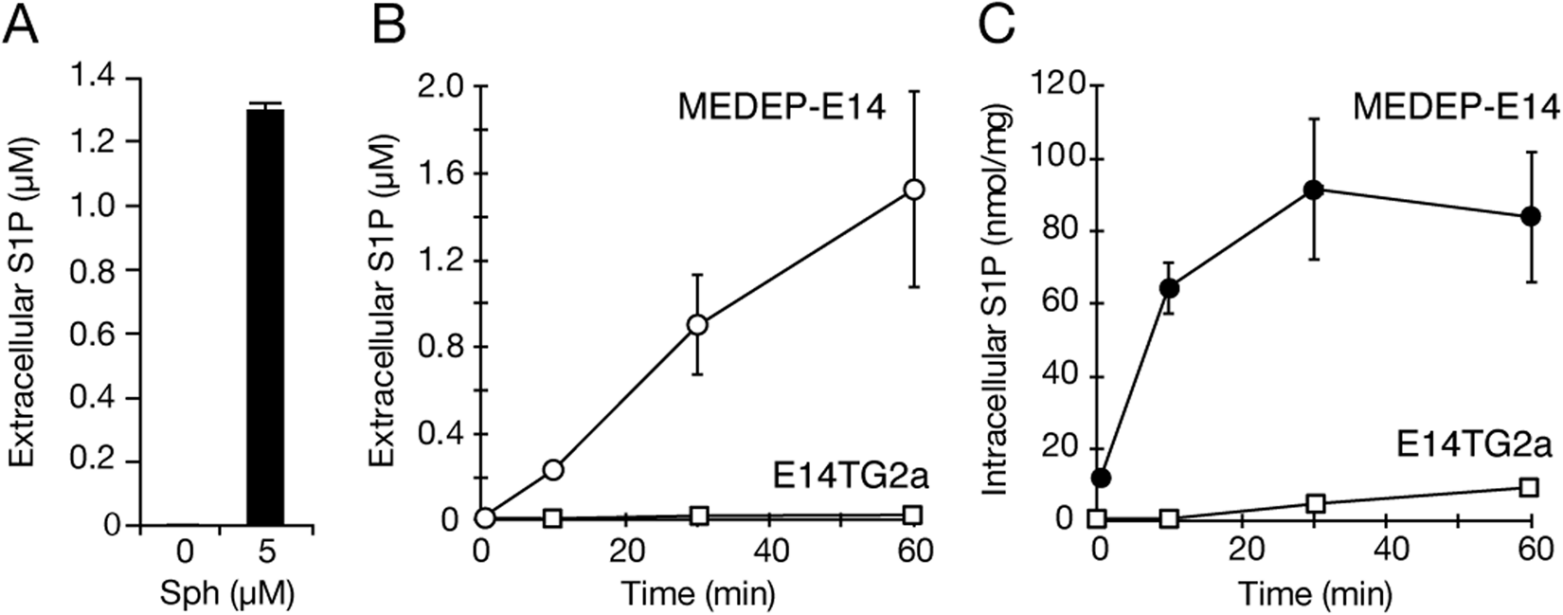
S1P synthesis and secretion in MEDEP-E14 and E14TG2a cells. (**A**) MEDEP-E14 cells (4 × 10^5^ cells) were incubated in the IMDM releasing medium with or without 5 μM sphingosine for two hours at 37 °C. The experiments were repeated three times (n = 3), and the error bars indicate the S.D. B, C. E14TG2a cells were incubated in the GMEM releasing medium containing 5 μM sphingosine at 37 °C for the indicated time. MEDEP-E14 cells (4 × 10^5^ cells) were incubated in the IMDM releasing medium containing 5 μM sphingosine for the indicated time at 37 °C. The amounts of S1P in the medium (extracellular, **B**) and cells (intracellular, **C**) were quantified. The experiments were repeated four times (n = 4), and the error bars indicate the S.D.

Together with these screening experiments, we searched erythrocyte model cell systems in which S1P is produced and released as observed in erythrocytes. We found a mouse erythroid cell line, MEDEP-E14, that has these activities. MEDEP-E14 cells were established through differentiation from the mouse embryonic stem cells E14TG2a^14^. MEDEP-E14 cells synthesized low levels of S1P endogenously, and thus, S1P in the medium was undetected without addition of an S1P precursor, sphingosine (Fig. 1A). However, when sphingosine was added to MEDEP-E14 cells, S1P was produced intracellularly and exported from the cells (Fig. 1A). In contrast to MEDEP-E14 cells, the S1P export activity was not observed in parental E14TG2a cells (Fig. 1B), probably because of very low S1P synthesis and/or export activity in E14TG2a cells (Fig. 1C). Extracellular addition of sphingosine to MEDEP-E14 cells increased the intracellular S1P concentration in a time-dependent manner, and the concentration reached a maximum at 30 min (Fig. 1C). In response to this increased intracellular S1P concentration, S1P was exported from the cells (Fig. 1B). These properties of S1P production and export in MEDEP-E14 cells are similar to those observed in erythrocytes^12^.

**Figure 2:**
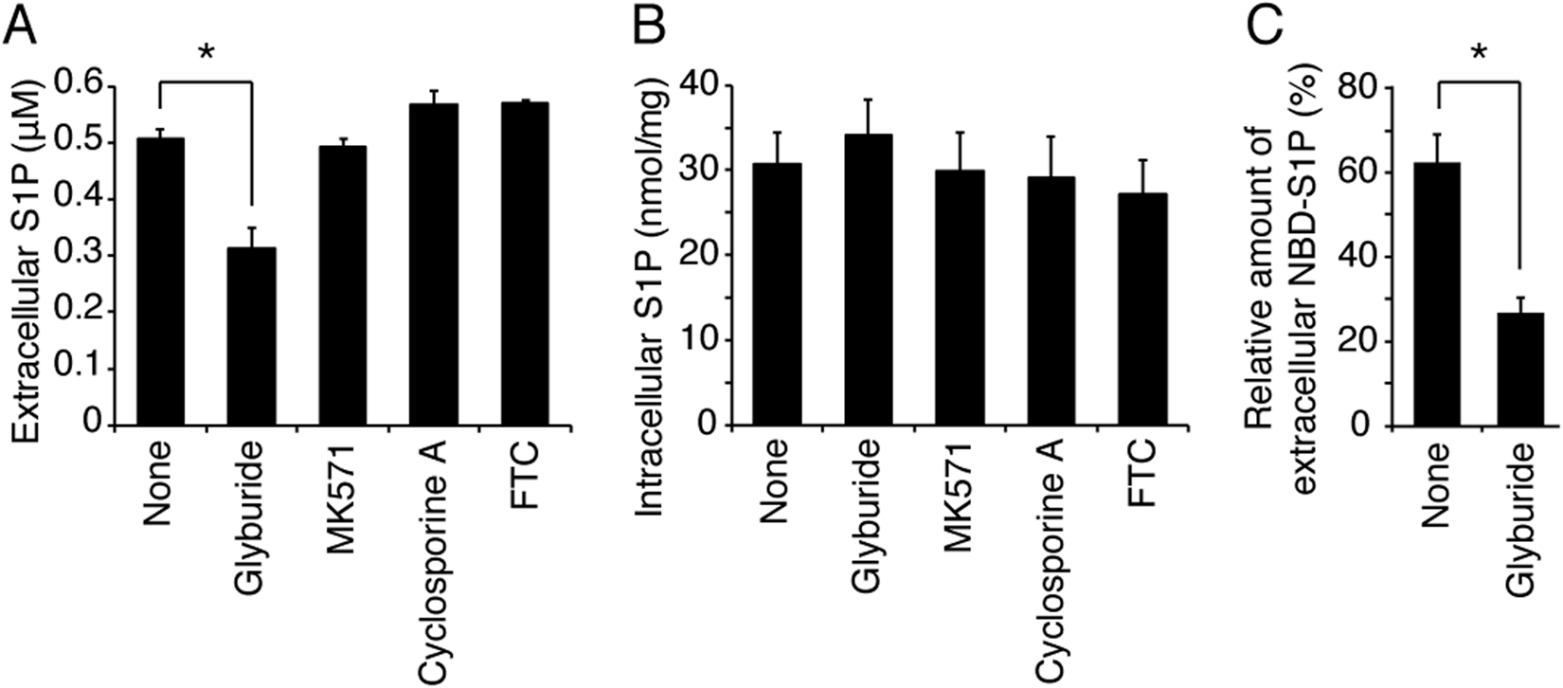
Effect of the erythrocyte S1P transporter inhibition on S1P and NBD-S1P export from MEDEP-E14 cells. (**A–C**) The assay was performed according to the procedures described in the materials and methods section, except that the concentration of BSA in the releasing medium was 0.1%. A, B. MEDEP-E14 cells (4 × 10^5^ cells) were pre-incubated in 190 μL of the IMDM releasing medium containing the indicated compounds at 37 °C for 10 min. Then, 10 μL of the IMDM releasing medium containing 100 μM sphingosine was added to the cell suspension. The final concentration of sphingosine, glyburide, MK571, Cyclosporine A and FTC were 5, 500, 50, 10 and 20 μM, respectively. After incubation at 37 °C for 30 min, the amount of S1P in the medium (**A**) and cells (**B**) was quantified. The experiments were repeated four times (n = 4), and the error bars indicate the S.D. Asterisk indicates a significant difference (P < 0.01; Tukey-Kramer test). (**C**) MEDEP-E14 cells (2 × 10^6^ cells) were pre-incubated in 94.5 μL of the IMDM releasing medium in the presence or absence of 500 μM glyburide at 37 °C for 10 min. Then, 5 μL of the IMDM releasing medium containing 100 μM NBD-sphingosine was added to the cell suspension. The final concentration of sphingosine was 5 μM. After incubation at 37 °C for 1 hr, the lipids in the cells and medium were extracted and analyzed by TLC. The amounts of NBD-S1P were quantified as described previously^15^. The relative amount of extracellular NBD-S1P was calculated by setting the total amount of NBD-S1P in the absence of inhibitor (none) to 100%. The experiments were repeated three times (n = 3), and the error bars indicate the S.D. Asterisk indicates a significant difference (P < 0.01; Welch’s t-test).

We previously reported that glyburide inhibits S1P export from erythrocytes^12^ and that a fluorescent S1P analogue, NBD-S1P, is exported from erythrocytes^15^. Thus, we examined whether S1P export from MEDEP-E14 cells shows the same characteristics of S1P transport activity observed in erythrocytes. Glyburide-treatment of MEDEP-E14 cells decreased S1P export from the cells (Fig. 2A) without altering the intracellular S1P concentration (Fig. 2B). In addition, NBD-S1P was also exported from MEDEP-E14 cells, and this export was inhibited by glyburide (Fig. 2C). These results strongly suggest that MEDEP-E14 expresses an erythrocyte-type S1P transporter and is a suitable model cell line to identify the erythrocyte S1P transporter because nucleated erythroid cells can be applied to molecular biology techniques.

### MFSD2B has S1P export activity

**Figure 3:**
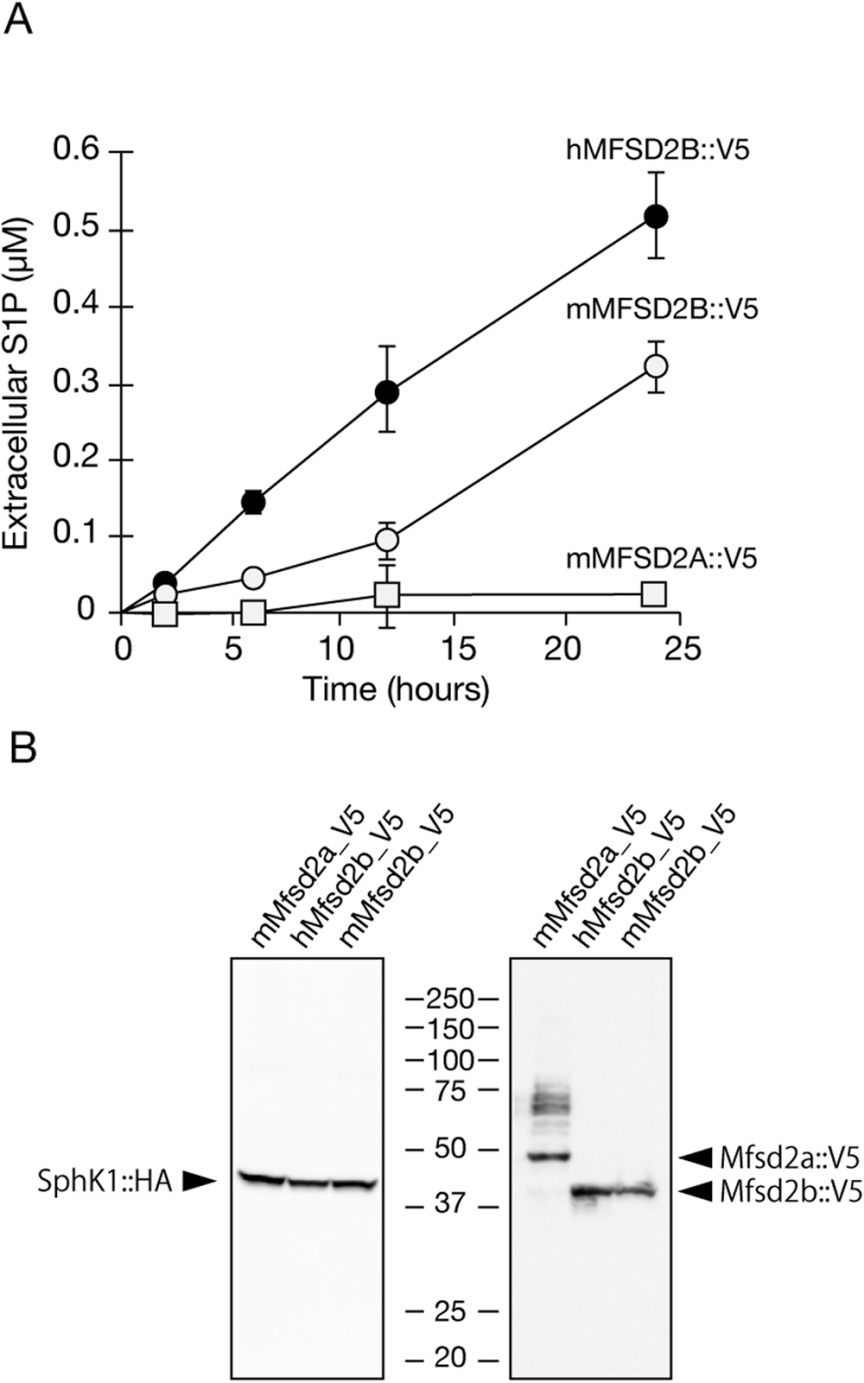
MFSD2B has the export activity of an endogenous S1P in cells. (**A**) CHO/SPHK1 cells stably expressing V5-tagged mouse MFSD2B (mMFSD2B::V5; open circle), human MFSD2B (hMFSD2B::V5; closed circle) or mouse MFSD2A (mMFSD2A::V5; closed square) were cultured in 6-well plates for two days. F12 releasing medium was added to the cells, which were then incubated for the indicated time periods. The amount of S1P in the releasing medium was determined as described in “Materials and Methods”. The experiments were repeated three times (n = 4), and the error bars indicate the S.D. (**B**) Membrane fractions from each of the transporters expressed in cells were isolated and subjected to Western blotting with anti-V5-HRP mAb. Expression of HA-tagged SPHK1 was detected with anti-HA mAb labeled with HRP and was used as a loading control for each sample.

Based on the evidence that S1P is exported in MEDEP-E14 cells but not in E14TG2a parental cells, we performed microarray analysis and selected three genes (*Slc28a2*, *Slc14a1* and *Mfsd2b*) encoding putative transporters that are more highly expressed in MEDEP-E14 cells than in E14TG2a cells (Supplemental Fig. 1A). We cloned each cDNA into an expression plasmid with either a V5- or PA-tag sequence at the 3′ end and then transfected the cloned constructs into CHO/SPHK1 cells. Expression of each transporter protein was detected by Western blotting of the V5- or PA-tagged proteins, and the S1P export activity of each transporter was measured. Neither SLC28A2 nor SLC14A1 exhibited any S1P export activity in the cells (Supplemental Fig. 1B,C), but MFSD2B showed export activity of endogenous S1P in a time-dependent manner (Fig. 3A). Mouse MFSD2B is a member of the major facilitator superfamily (MFS) of transporters and has 494 amino acid residues (Supplemental Fig. 2). *Mfsd2b* cDNA has been reported in various mammals. Human MFSD2B shows 83% identity and 96% similarity with mouse MFSD2B and exported endogenous S1P from CHO/SPHK1 cells (Fig. 3A). Both human and mouse MFSD2B proteins were detected as a band with a similar molecular size of approximately 43 kDa (Fig. 3B).

The MFSD2B homologue, MFSD2A, has been identified as a lysophosphatidylcholine transporter in endothelial cells of the blood-brain barrier^16^. The protein sequence for mouse MFSD2A showed relatively high similarity to that for mouse MFSD2B (42% identity and 79% similarity) (Supplemental Fig. 2). However, mouse MFSD2A could not export S1P from the cells (Fig. 3), which is consistent with a previous study in which MFSD2A did not uptake S1P into cells^16^. Although SPNS2 has been identified as the S1P transporter in endothelial cells by us and other groups, MFSD2B has less than 20% similarity to SPNS2 when comparing overall protein sequences (data not shown).

**Figure 4:**
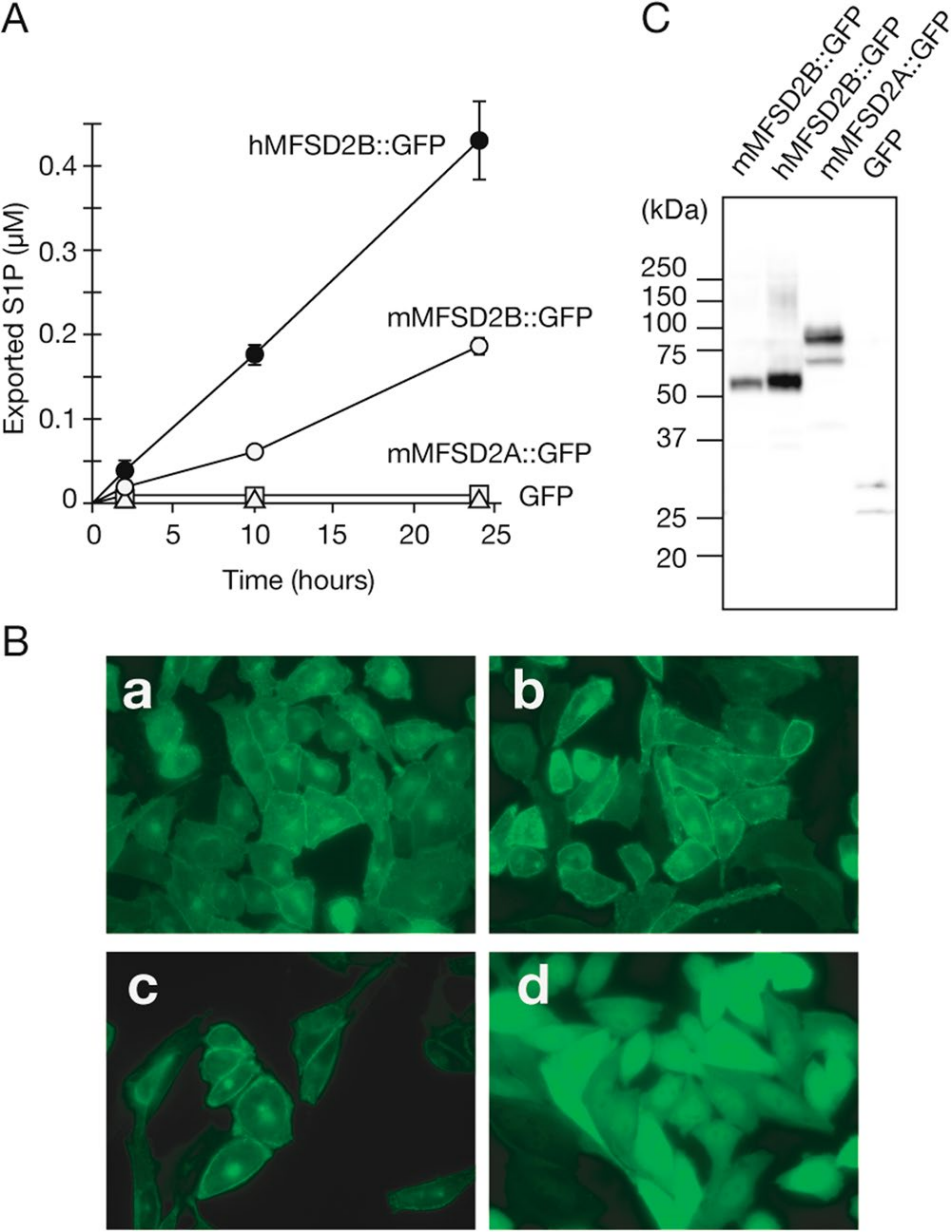
Localization and S1P export activity of GFP-tagged MFSD2B in cells. (**A**) CHO/SPHK1 cells stably expressing GFP-tagged mouse MFSD2B (mMFSD2B::GFP; open circle), human MFSD2B (hMFSD2B::GFP; closed circle), mouse MFSD2A (mMFSD2A::GFP; open square) or GFP alone (open triangle) were cultured in 6-well plates for two days. (**A)** Export activity of endogenous S1P in the cells was measured. The F12 releasing medium was added to the cells, and the cells were incubated for the indicated time. The amount of S1P in the releasing medium was determined as described in “Materials and Methods”. The experiments were repeated three times (n = 3), and the error bars indicate the S.D. (**B**) Fluorescent images of GFP-tagged mouse MFSD2B (**a**), human MFSD2B (**b**), mouse MFSD2A (**c**) or GFP alone (**d**) expressing cells were obtained by fluorescence microscopy. (**C**) Membrane fractions from each transporter expressed in cells were isolated and subjected to Western blotting with an anti-GFP mAb.

To evaluate the effects of tagging on the activity and cellular localization of MFSD2B, we also expressed GFP-tagged MFSD2B in CHO/SPHk1 cells (Fig. 4). Human and mouse constructs of GFP- and V5-tagged MFSD2B showed similar S1P export activity, suggesting that carboxyl-terminal tagging of the proteins did not influence the S1P transport activity of MFSD2B (Figs 3A and 4A). Similar to the SPNS2 protein, the GFP-tagged mouse and human MFSD2B proteins were localized to the plasma membrane of the cells (Fig. 4B). GFP-tagged mouse MFSD2A was also localized to the plasma membrane but did not show S1P export activity (Fig. 4B). The molecular size of these proteins in Western blotting was increased by GFP tagging (approximately 28 kDa) (Fig. 4C). A higher level of S1P export activity was observed in the cells expressing human MFSD2B than in the cells expressing mouse MFSD2B, correlating with the protein expression levels of these proteins (Fig. 4B,C).

**Figure 5:**
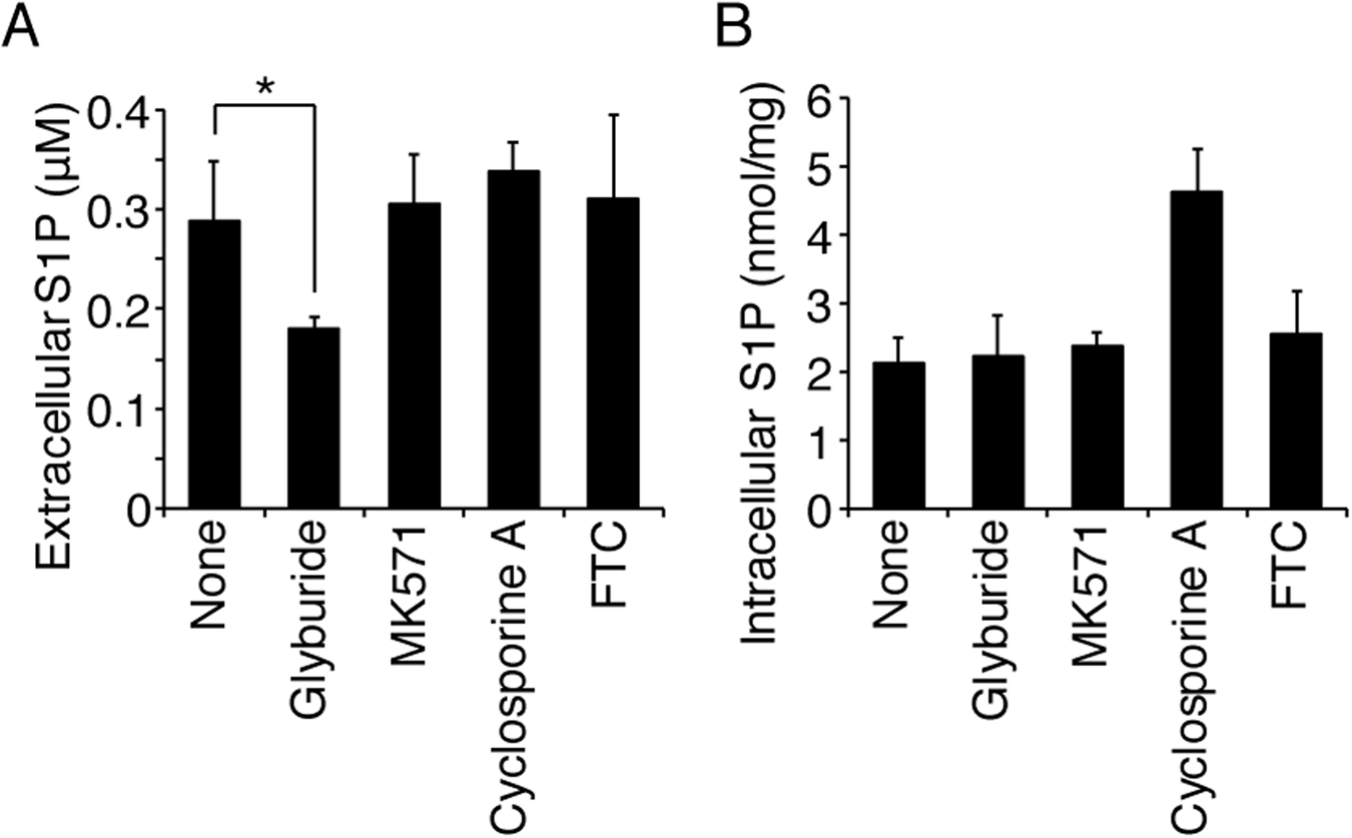
Effect of the erythrocyte S1P transporter inhibitor on S1P export activity of mouse MFSD2B. (**A**,**B**) CHO/SPHK1 cells stably expressing V5-tagged mouse MFSD2B were cultured in 6-well plates for two days. The F12 releasing medium containing 5 µM sphingosine and each transporter inhibitor was added to the cells. The final concentrations of glyburide, MK571, cyclosporine A and FTC were 500, 50, 10 and 20 µM, respectively. After incubation at 37 °C for two hours, the amount of S1P in the medium (**A**) or cells (**B**) was quantified. The experiments were repeated four times (n = 4), and the error bars indicate the S.D. Asterisk indicates a significant difference (P < 0.05; Tukey-Kramer test).

We showed that properties of the S1P export activity of MEDEP-E14 cells is similar to that of erythrocytes (Figs 1 and 2). It is of interest to determine whether MFSD2B operates as an S1P transporter in both MEDEP-E14 cells and erythrocytes. As shown in Fig. 5, the S1P export activity of mouse MFSD2B was partially inhibited by glyburide but not by other inhibitors. This inhibition profile in S1P transport activity of MFSD2B resembles that observed for MEDEP-E14 cells and erythrocytes (Figs 2 and 5)^12^.

### S1P transport activity of MFSD2B is not sodium dependent

**Figure 6:**
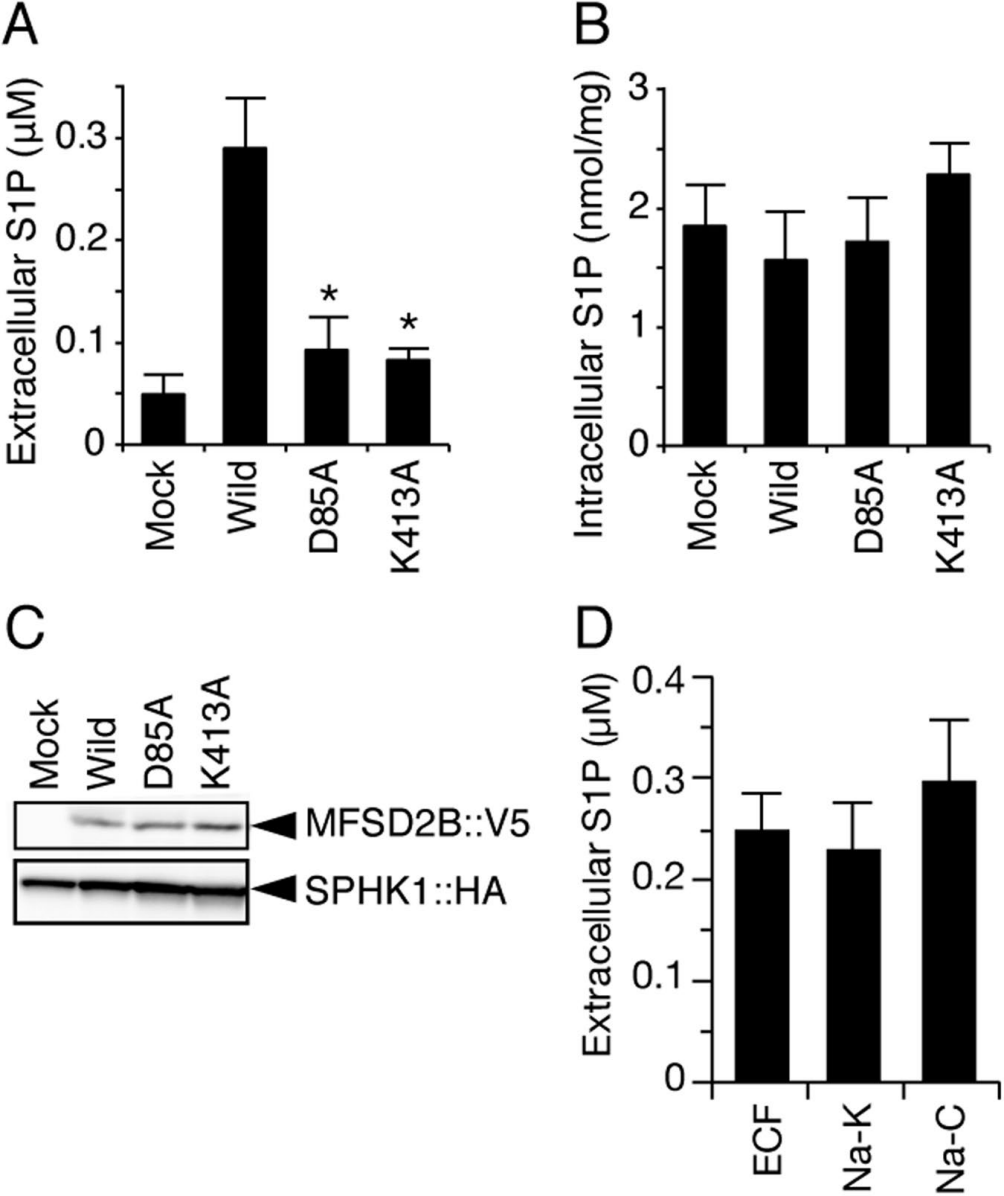
S1P export activity from CHO/SPHK1 cells expressing mouse MFSD2B mutants. (**A**,**B**) CHO/SPHK1 cells stably expressing V5-tagged wild-type (Wild) or mutant (D85A or K413A) mouse MFSD2B were cultured in 6-well plates for two days. CHO/SPHK1 cells containing only the expression vector was used for a control (Mock). F12 releasing medium containing 5 µM sphingosine was added to the cells. After incubation at 37 °C for two hours, the amount of S1P in the medium (**A**) or cells (**B**) was quantified. The experiments were repeated three times (n = 6), and the error bars indicate the S.D. Asterisks indicate significant differences (P < 0.01 vs. wild-type; Tukey-Kramer test). (**C**) CHO/SPHK1 cells stably expressing V5-tagged wild-type mouse MFSD2B (wild) or mutated mouse MFSD2B (D85 A or K413A) were analyzed by Western blotting with an anti-V5-HRP mAb. Expression of HA-tagged SPHK1 was detected with an anti-HA mAb labeled with HRP and was used for a loading control for each sample. A full-length blot image is shown in Supplemental Fig. 5A. (**D**) CHO/SPHK1 cells stably expressing V5-tagged mouse MFSD2B were cultured in 6-well plates for two days. ECF releasing medium (ECF), releasing medium in which Na^+^ was replaced with K^+^ (Na-K), or releasing medium in which Na^+^ was replaced with choline (Na-Choline) containing 5 µM sphingosine was added to the cells. After incubation at 37 °C for two hours, the amount of S1P in the medium was quantified. The experiments were repeated three times (n = 3), and the error bars indicate the S.D.

A previous mutagenesis study of human MFSD2A suggested participation of Asp93 in sodium binding and Lys436 in interaction with a phosphate head group of substrates^17^. In the homology model of MFSD2A, Asp93 and Lys436 are located in close proximity^17^. When comparing the amino acid sequences of human MFSD2A and mouse MFSD2B, Asp93 is not conserved in MFSD2B (Supplemental Fig. 2). However, Asp97 and Lys436 residues of MFSD2A are conserved between the two proteins, and these residues correspond to Asp85 and Lys413 residues of mouse MFSD2B (Supplemental Fig. 2). Asp97 is one of the residues constituting the sodium-binding site of human MFSD2A^17^. Single alanine substitution mutants at Asp85 and Lys413 of mouse MFSD2B were expressed in CHO/Sphk1 cells, and the S1P export activities of those mutants were measured (Fig. 6). Both mutants showed a basal level of activity of S1P export (Fig. 6A) without affecting the intracellular S1P concentration (Fig. 6B) or MFSD2B protein expression (Fig. 6C), indicating that these residues are important for S1P export activity of MFSD2B. Because Asp residues are generally reported to constitute ion binding sites, such as for the Na^+^ or K^+^ ion, and to be important for the coupling of substrate transport and cation flux in various membrane transporters^18,19^, it is possible that S1P transport by MFSD2B also couples to Na^+^ or K^+^ flux.

To explore this possibility, we used the release medium, in which Na^+^ was substituted with K^+^ or choline, for measurement of S1P export activity in the MFSD2B-expressing cells. As shown in Fig. 6D, disruption of the ion gradient by substitution of Na^+^ with K^+^ or choline in the release medium did not affect the S1P export activity in MFSD2B-expressing cells. These results suggest that S1P export activity of MFSD2B is not driven by a Na^+^ or K^+^ ion gradient, although alanine substitution of Asp85 affected the S1P transport of MFSD2B, which is consistent with observations for the corresponding residue of MFSD2A.

### MFSD2B is an S1P transporter in erythroid cells

**Figure 7:**
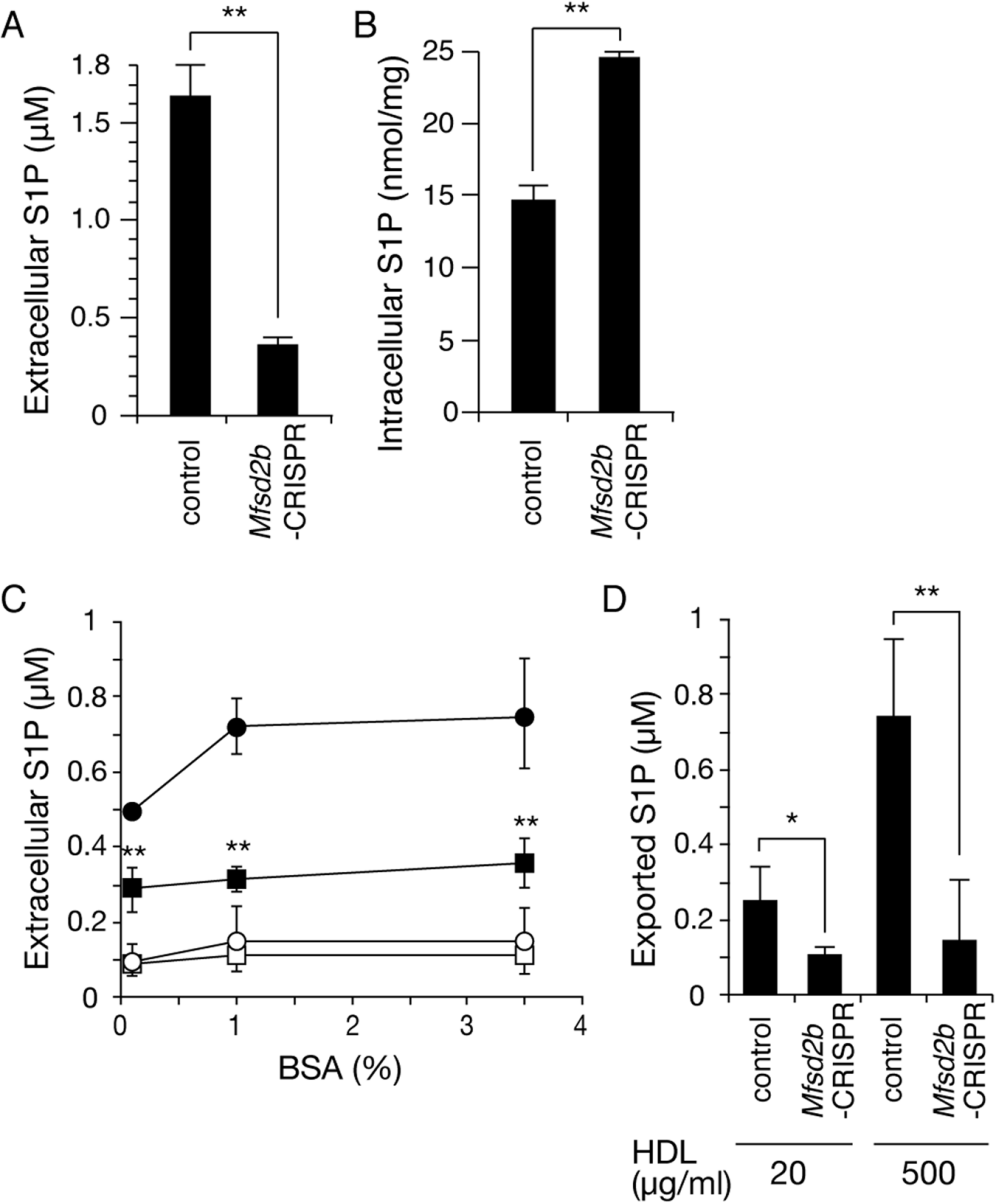
Loss-of-function analysis of MFSD2B using the CRISPR/Cas9 system. (**A**,**B**) MEDEP-E14 cells infected with lentivirus encoding random DNA control (control) or lentivirus encoding Cas9-gRNA targeting *Mfsd2b* (CRISPR-*Mfsd2b*) (4 × 10^5^ cells) were incubated in IMDM releasing medium with 5 μM sphingosine for two hours at 37 °C. The amounts of S1P in the medium (**A**) and cells (**B**) were quantified. The experiments were repeated four times (n = 5), and the error bars indicate the S.D. BSA (**C**) or HDL (**D**) concentration-dependent S1P export from MEDEP-E14 control and CRISPR- *Mfsd2b* cells was measured. The cells were pre-incubated in IMDM containing 0.1% BSA and 5 μM sphingosine at 37 °C for 10 min. Then, the cells were washed once with IMDM and incubated with IMDM containing different concentrations of BSA (0.1, 1, and 3.5%) or HDL (20 and 500 μg/ml) at 37 °C for 30 min. The amounts of S1P in the medium were quantified. (**C**) Open and closed circles indicate the extracellular S1P concentration of MEDEP-E14 control cells at 0 min and 30 min, respectively. Open and closed squares indicate the extracellular S1P concentration of MEDEP-E14 CRISPR- *Mfsd2b* cells at 0 min and 30 min, respectively. The experiments were repeated four times (n = 4), and the error bars indicate the S.D. (**D**) Amount of HDL-dependent export was determined by subtraction of the amount of extracellular S1P at 0 min from that at 30 min. The experiments were repeated four times (n = 4), and the error bars indicate the S.D. Asterisk indicate a statistically significant difference (*P < 0.05, **P < 0.01; Welch’s t-test) against MEDEP-E14 control.

To confirm that MFSD2B is an S1P transporter in MEDEP-E14 cells, loss-of-function analysis was performed using the CRISPR/Cas9 system. As shown in Fig. 7A, in *Mfsd2b*-CRISPR MEDEP-E14 cells, S1P export activity was significantly decreased to 20% of that in the wild-type cells. Consistent with the decrease of S1P export activity, amount of intracellular S1P of the *Mfsd2b*-CRISPR MEDEP-E14 cells was increased compared to the control MEDEP-E14 cells (Fig. 7B). We also examined the BSA concentration dependency of S1P export from MEDEP-E14 cells up to 3.5% (3.5 g/dL) BSA, which is the physiological concentration of serum albumin (Fig. 7C). The amount of extracellular S1P increased depending on the increase in extracellular BSA concentration up to 1%. In blood plasma, although approximately 35% of S1P is bound to albumin, about 60% of S1P is bound to high-density lipoprotein (HDL)^20^. Just before the submission of our manuscript, Christensen *et al*. reported that HDL and apolipoprotein M (ApoM) are good carriers for S1P exported from erythrocytes^21^. As shown in Fig. 7D, the amounts of exported S1P from MEDEP-E14 cells using the physiological concentration of HDL (500 μg/ml) in releasing medium were comparable to those when using 1% (1 g/dL) BSA (Fig. 7C). Furthermore, S1P export activity was significantly decreased in the *Mfsd2b*-CRISPR MEDEP-E14 cells at any concentration of BSA or HDL (Fig. 7C,D). These results strongly support the hypothesis that MFSD2B is the S1P transporter in MEDEP-E14 cells.

### Detection of *Mfsd2b* transcripts in mouse tissues and MFSD2B protein in erythroid cells

**Figure 8:**
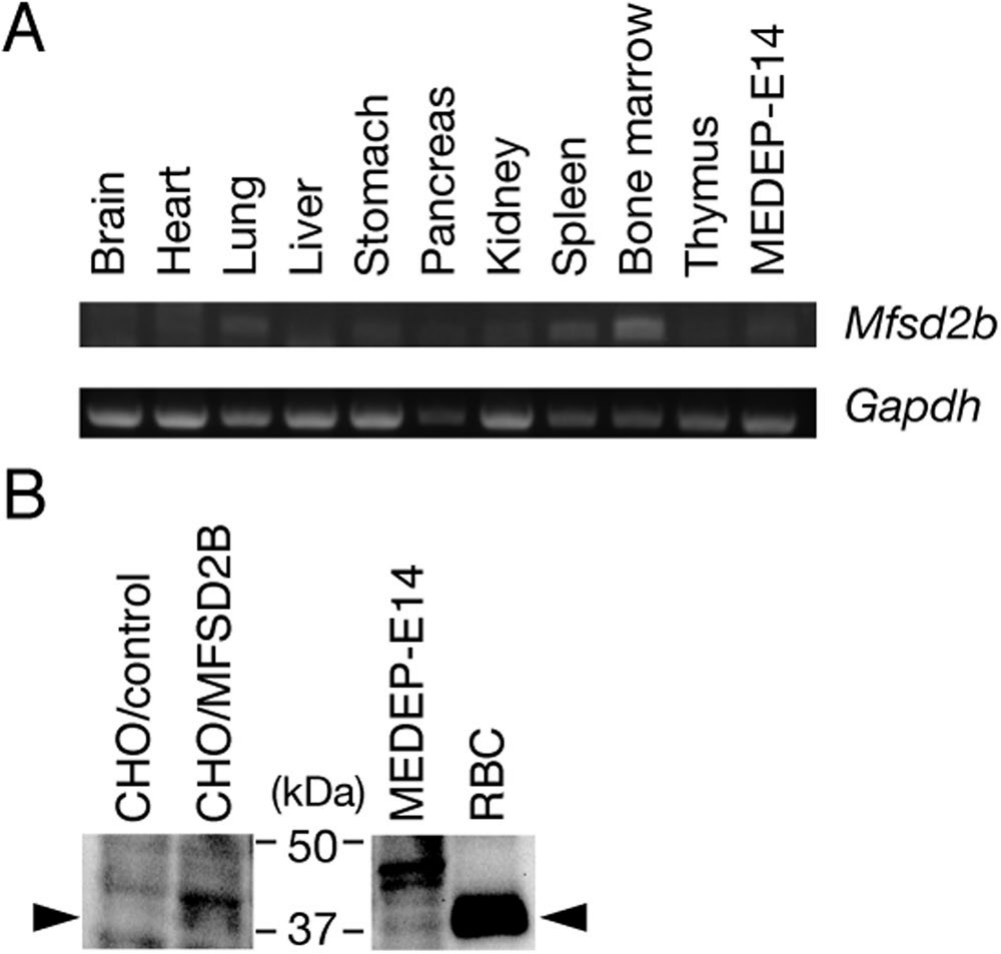
Tissue distribution of mouse *Mfsd2b* mRNAs and expression of mouse Mfsd2b in erythroid cells. (**A**) Total RNA isolated from various mouse tissues and MEDEP-E14 cells was subjected to RT-PCR. Amplified products of *Mfsd2b* and *Gapdh* were analyzed by 1.5% agarose gel electrophoresis. Full-length gel images were indicated in Supplemental Fig. 6. (**B**) Expression of MFSD2B protein in the mock-transfected CHO/SPHK1 cells (CHO/control), CHO/SPHK1 cells stably expressing MFSD2B (CHO/MFSD2B), MEDEP-E14 cells and mouse erythrocytes (RBC) was detected by Western blotting with rabbit anti-mouse MFSD2B polyclonal antibodies. Full-length blot image was indicated in Supplemental Fig. 5B.

*In vitro* analysis using MEDEP-E14 cells demonstrated that MFSD2B is the S1P transporter in erythroid cells. Next, we examined tissue distribution of *Mfsd2b* in mouse tissues by RT-PCR analysis. *Mfsd2b* transcripts were mainly detected in bone marrow and weakly detected in all tissues (Fig. 8A), which may result from production of major erythrocytes in bone marrow and the presence of a few erythroid cells in all tissues throughout the blood stream. These results suggest that MFSD2B is the S1P transporter in erythroid cells, such as erythrocytes. Supporting this possibility, expression of MFSD2B protein in mouse erythrocytes was confirmed by immunoblotting with anti-MFSD2B antibodies (Fig. 8B).

## Discussion

Erythrocytes and vascular endothelial cells play important roles in maintaining a constitutive S1P concentration in blood plasma. While S1P released from endothelial cells via SPNS2 regulates lymphocyte egress from thymus and secondary lymphoid organs to the blood^7,22–25^, the S1P transporter has not been identified in erythrocytes, and its physiological roles have been unclear. Although the S1P transporter is essential for S1P release from erythrocytes, efforts to identify the S1P transporter by genetic manipulation have been difficult because erythrocytes have no nucleus. In this study, we found that a nucleated erythroid cell line, MEDEP-E14, takes up extracellular sphingosine, produces S1P intracellularly, and releases S1P to the extracellular space without any external stimulations, which is suppressed by glyburide (Figs 1 and 2). These properties are similar to those observed in erythrocytes^12^. By performing microarray analysis of MEDEP-E14 cells, we succeeded in identifying a novel S1P transporter, MFSD2B. When MFSD2B was expressed in CHO/SPHK1 cells, intracellular S1P was released extracellularly in a stimulus-independent manner, similar to that observed in MEDEP-E14 cells (Figs 1 and 3). The properties of this S1P release are similar to those in SPNS2-expressing CHO/SPHK1 cells. SPNS2 is the S1P transporter expressed in vascular endothelial cells^7^. Both SPNS2 and MFSD2B are types of MFS transporters^24,26^. However, SPNS2 and MFSD2B share less than 8% identity and 44% similarity in amino acid sequence. Molecular modeling of SPNS2 and MFSD2B using crystal structures of the bacterial MFS transporters, GlpT^27^ and MelB^18^, respectively, showed that both transporters have twelve trans-membrane regions (TMs) (Supplemental Figs 2 and 3). Arg200 in TM7 of SPNS2 is essential for S1P transport^28^ and is located on the cytoplasmic side of the plasma membrane near the interface of protein and membrane lipids where S1P could have access to the Arg200 residue horizontally from the membrane (Supplemental Fig. 3)^8^. Lys413 and Asp85 of MFSD2B are also important for S1P transport activity and are located in the middle of the protein in close proximity to the membrane lipids (Supplemental Fig. 3). Although there is not significant sequence homology between SPNS2 and MFSD2B, these properties of the charged residues suggest that the charged residues of these transporters may be involved in the binding or recognition of S1P and are located near the interface of transmembrane helices and the plasma membrane.

Previously, the ABC transporters, ABCA1, ABCC1, ABCB1, and ABCG2, were reported to transport S1P or its analog (that is, the phosphorylated form of FTY720) in various types of cells^29–35^. However, when these ABC transporters were expressed in CHO/SPHK1 cells, in which SPNS2 and MFSD2B function as an S1P transporter, the ABC transporters did not export S1P on their own^13^. As shown in Figs 2 and 5, the S1P export from MEDEP-E14 cells and MFSD2B-expressing cells was inhibited by glyburide but not by MK571, Cyclosporine A, or FTC, which are known to inhibit ABCA1, ABCC1, ABCB1, and ABCG2, respectively. Lee *et al*. have previously shown that the plasma S1P level in ABCA1, ABCA7, or ABCC1 knock-out mice was not altered^36^. Furthermore, we showed that ABCA1 protein is not detectable in rat erythrocyte membranes^12^ and that the S1P export activity of erythrocytes and platelets isolated from ABCA7 knock-out mice is comparable to that in wild-type mice^13^. Taken together, these results suggest that these ABC transporters do not contribute to the S1P export from erythroid cells.

Recently, Christensen *et al*. showed that S1P export from erythrocytes to ApoM was inhibited by an ABCC1 inhibitor, MK571, although S1P is normally released from erythrocytes to ApoM in ABCC1 knock-out mice^21^. This is consistent with the previous observation that the S1P concentration in the blood plasma of ABCC1 knock-out mice is not altered^36^. We previously showed that the S1P transport from rat erythrocytes to BSA is not inhibited by MK571^12^. In the current study, the S1P export activities using BSA (1%; 1 g/dL) and HDL (500 μg/ml) were comparable (Fig. 7C,D). Although 1% BSA is a lower concentration than that of normal serum albumin (3.5–5.5%; g/dL), 1% BSA has sufficient ability to bind about 1 μM S1P in the medium (Fig. 7). Similar results were observed for NBD-S1P export from the MEDEP-E14 cells (Supplemental Fig. 4). Because the plasma S1P concentration is maintained at about 1 μM, BSA, together with HDL, should be an appropriate carrier for the *in vitro* assay of S1P transporter activity.

During the preparation of this manuscript, analysis of *Mfsd2b* knockout mice was reported^37^. The *Mfsd2b* knockout mice showed a decrease in plasma S1P concentration and defective S1P export activity in erythrocytes^37^. The results from *Mfsd2b* knockout mice and our erythroid model cells together strongly indicate that MFSD2B plays a central role in S1P export from erythroid cells.

The similarity in the properties of S1P release from MEDEP-E14 cells and erythrocytes indicates that S1P release from MEDEP-E14 cells is a suitable model for analyzing S1P export from erythrocytes. Because application of the CRISPR/Cas9 system targeted to the *Mfsd2b* gene in MEDEP-E14 cells dramatically decreased S1P export activity, nucleated MEDEP-E14 cells may be useful for both analyzing MFSD2B function and screening inhibitors of MFSD2B.

## Material and Methods

### Reagents

BSA (bovine serum albumin, fatty acid-free; # A6003), human high density lipoprotein (HDL; # L1567), and sphingosine (Sph) were obtained from Sigma. S1P, C_17_-sphingosine 1-phosphate (C_17_-S1P) and NBD-sphingosine were purchased from Avanti. The anti-V5-tag mouse monoclonal antibody conjugated to HRP was purchased from Invitrogen. The anti-GFP rabbit polyclonal antibody was purchased from MBL. All other chemicals were of analytical or HPLC grade and were obtained from commercial sources.

### Cell culture

E14TG2a cells^38^ and MEDEP-E14 cells^14^ were obtained from RIKEN Cell Bank. E14TG2a cells were cultured in dishes coated with gelatin (Wako Pure Chemical) and maintained in Glasgow’s minimum essential medium (GMEM, Wako Pure Chemical) supplemented with 10% fetal bovine serum (GE Healthcare), 0.1 mM non-essential amino acids (Wako Pure Chemical), 1 mM sodium pyruvate (Wako Pure Chemical), 1000 U/mL mouse leukemia inhibitory factor (Merck Millipore) and 0.1 mM 2-mercaptoethanol (Wako Pure Chemical). MEDEP-E14 cells were maintained in Iscove’s modified Dulbecco’s medium (IMDM) containing 2 mM L-glutamine (Wako Pure Chemical) supplemented with 15% fetal bovine serum (GE Healthcare), 10 mg/ml bovine insulin, 5.5 mg/ml human transferrin, 5 ng/ml sodium selenite (ITS Liquid Media Supplement, Sigma), 50 mg/ml ascorbic acid (Sigma), 0.45 mM α-monothioglycerol (Sigma), and 3 unit/ml human erythropoietin (Kyowa Hakko Kirin).

### Construction of transporter-expressing cell lines

The cDNA of mouse *Mfsd2b* (NM_001033488), mouse *Mfsd2a* (NM_029662), and human *Mfsd2b* (NM_001346880) was synthesized (Eurofins genomics). Each cDNA fragment was cloned into the pcDNA5/FRT vector, and the sequences were confirmed by DNA sequencing. All of the constructed plasmids were individually transfected into Flp-In-CHO/SPHK1 cells to establish CHO cell lines stably expressing both SPHK1 and each transporter as described previously^13^. The cell lines expressing each transporter were selected by treatment with hygromycin B.

### RT-PCR

Total RNA was extracted from cells using the PureLink RNA Mini Kit (Invitrogen) according to the manufacturer’s instructions. The concentration and purity of the RNA were determined spectrophotometrically by measuring the absorbance at 260 nm and 280 nm using a NanoDrop (Thermo). The mRNA was reverse transcribed using the SuperScript III cDNA Synthesis Kit (Invitrogen). For amplification of the mouse *Mfsd2b gene*, we used primers (Mfsd2b-Fw; 5′-ccttaatcgcactggcctacttct-3′ and Mfsd2b-Rv; 5′-caaccacggcagctgcaatacaat-3′) and first-strand cDNA prepared from C57BL/6 J mouse tissues, which was obtained from Genostaff Co., Ltd. Glyceraldehyde-3-phosphate dehydrogenase cDNA (*Gapdh*) was used as a control and was amplified with following primers: Gapdh-Fw, 5′-tgaaggtcggtgtgaacggatttggc-3′ and Gapdh-Rv, 5′-catgtaggccatgaggtccaccac-3′.

### Microarray analysis

E14TG2a and MEDEP-E14 cells were collected by centrifugation. Buffer RLT (QIAGEN) was added to the cells and the cells were lysed by vortex mixing. The lysed cells were frozen in liquid nitrogen. Extraction of total RNA, preparation of cRNA and microarray analysis were performed by a commercial microarray service (Bio Matrix Research, Nagareyama, Japan). RNA was extracted by using an RNeasy Mini Kit (QIAGEN). The RNA quality was evaluated using an Agilent 2100 Bioanalyzer (Agilent Technologies) and NanoDrop ND-1000 (NanoDrop Technologies). cRNA was synthesized according to the protocol of a Low Input Quick Amp Labeling Kit (Agilent Technologies) from 100 ng of total RNA. cRNA was labeled by using Cyanine 3 labeled-CTP. The labeled cRNA was hybridized on a SurePrint G3 Mouse GE Microarray (8 × 60 K) for 17 hours and then washed and scanned using a Microarray Scanner (Agilent Technologies). The fluorescence intensity of each spot on the microarray was quantified by Feature Extraction Software (Version 10.7, Agilent Technologies). The quantified raw data were analyzed by using GeneSpring GX software (Agilent Technologies). Normalization of the data was performed using percentile shift (85th percentile). Genes that were determined to be present in MEDEP-E14 cells but absent in E14TG2a cells are listed in Supplemental Fig. 1A.

### S1P transport assay

The S1P transport assay was performed according to a modified protocol of Hisano *et al*.^13^ MEDEP-E14 cells were cultured in 15 ml flasks and suspended at approximately 1 × 10^5^ cells/ml. E14TG2a cells or CHO/SPHK1 cells, which were transfected with various transporter-expressing plasmids, were cultured in a 24-well plate until the number of cells reached approximately 2 × 10^5^ cells/ml.

The S1P amounts in the samples were quantified by HPLC analysis following *o*-phthalaldehyde (OPA) modification of the lipids as described previously^39^. Briefly, the cells were washed twice in cell culture medium without FBS and incubated with sphingosine in the releasing medium (cell culture medium containing 1% BSA, 10 mM sodium glycerophosphate, 5 mM sodium fluoride and 1 mM semicarbazide) at 37 °C. After the incubation, 100 μL aliquots of the medium were transferred to new tubes and subjected to lipid extraction under alkaline chloroform conditions. C_17_-S1P (30 pmol) was added to each sample as the internal standard. Extracted S1P was dephosphorylated with calf intestinal alkaline phosphatase at 37 °C for 90 min. The resulting Sph was extracted with chloroform. Then, chloroform was dried, and Sph was dissolved in ethanol. OPA modification of Sph was performed at room temperature for 1 hour, and 10 μL of each sample was analyzed by HPLC (Hitachi) using a Cosmosil 5 C 18-AR-II column (Nacalai tesque) and a fluorescence detector.

### Western blotting

Cells were sonicated in PBS supplemented with a protease inhibitor mixture (Nacalai). 30 μg of proteins were separated using a 12.5% SDS-PAGE gel, and transferred to a nitrocellulose membrane. Tagged and native transporter proteins were detected by immunoblotting with specific antibodies against them. Rabbit anti-mouse MFSD2B polyclonal antibodies were generated using the synthetic peptide corresponding to the amino terminal region (amino acids 15 to 27) of the mouse MFSD2B protein.

### Statistics

The experiments were performed independently at least three times, and each experiment was carried out in single or duplicate sample(s). The error bars indicate the standard deviation (S.D.) of all measurement data in each experimental condition. To analyze statistical significance, we used unpaired two-tailed t-tests with Welch’s correction for comparisons of two groups or Tukey-Kramer tests for multiple comparisons (JMP Pro statistical software, version 13.1; SAS Institute). We considered P-values < 0.05 to be significant.

### Data availability

The data used in this article are available from the corresponding author.

## Electronic supplementary material

Supplemental Figures

## References

[1] Spiegel S, Milstien S (2011). The outs and the ins of sphingosine-1-phosphate in immunity. Nature reviews. Immunology.

[2] Cyster JG, Schwab SR (2012). Sphingosine-1-phosphate and lymphocyte egress from lymphoid organs. Annu Rev Immunol.

[3] Proia RL, Hla T (2015). Emerging biology of sphingosine-1-phosphate: its role in pathogenesis and therapy. J Clin Invest.

[4] Ishii M (2009). Sphingosine-1-phosphate mobilizes osteoclast precursors and regulates bone homeostasis. Nature.

[5] Blaho VA, Hla T (2014). An update on the biology of sphingosine 1-phosphate receptors. J Lipid Res.

[6] Yanagida K, Hla T (2017). Vascular and Immunobiology of the Circulatory Sphingosine 1-Phosphate Gradient. Annu Rev Physiol.

[7] Hisano Y, Kobayashi N, Yamaguchi A, Nishi T (2012). Mouse SPNS2 Functions as a Sphingosine-1-Phosphate Transporter in Vascular Endothelial Cells. PloS one.

[8] Nishi T, Kobayashi N, Hisano Y, Kawahara A, Yamaguchi A (2014). Molecular and physiological functions of sphingosine 1-phosphate transporters. Biochim Biophys Acta.

[9] Pappu R (2007). Promotion of lymphocyte egress into blood and lymph by distinct sources of sphingosine-1-phosphate. Science.

[10] Yang L, Yatomi Y, Miura Y, Satoh K, Ozaki Y (1999). Metabolism and functional effects of sphingolipids in blood cells. Br J Haematol.

[11] Kihara A, Igarashi Y (2008). Production and release of sphingosine 1-phosphate and the phosphorylated form of the immunomodulator FTY720. Biochim Biophys Acta.

[12] Kobayashi N, Kobayashi N, Yamaguchi A, Nishi T (2009). Characterization of the ATP-dependent sphingosine 1-phosphate transporter in rat erythrocytes. J Biol Chem.

[13] Hisano Y, Kobayashi N, Kawahara A, Yamaguchi A, Nishi T (2011). The sphingosine 1-phosphate transporter, SPNS2, functions as a transporter of the phosphorylated form of the immunomodulating agent FTY720. J Biol Chem.

[14] Hiroyama T (2008). Establishment of mouse embryonic stem cell-derived erythroid progenitor cell lines able to produce functional red blood cells. PloS one.

[15] Kobayashi N, Otsuka M, Yamaguchi A, Nishi T (2016). Fluorescence-based rapid measurement of sphingosine-1-phosphate transport activity in erythrocytes. J Lipid Res.

[16] Nguyen LN (2014). Mfsd2a is a transporter for the essential omega-3 fatty acid docosahexaenoic acid. Nature.

[17] Quek DQ, Nguyen LN, Fan H, Silver DL (2016). Structural Insights into the Transport Mechanism of the Human Sodium-dependent Lysophosphatidylcholine Transporter MFSD2A. J Biol Chem.

[18] Ethayathulla AS (2014). Structure-based mechanism for Na( + )/melibiose symport by MelB. Nat Commun.

[19] Rosental N, Gameiro A, Grewer C, Kanner BI (2011). A conserved aspartate residue located at the extracellular end of the binding pocket controls cation interactions in brain glutamate transporters. J Biol Chem.

[20] Murata N (2000). Interaction of sphingosine 1-phosphate with plasma components, including lipoproteins, regulates the lipid receptor-mediated actions. Biochem J.

[21] Christensen PM, Bosteen MH, Hajny S, Nielsen LB, Christoffersen C (2017). Apolipoprotein M mediates sphingosine-1-phosphate efflux from erythrocytes. Sci Rep.

[22] Fukuhara S (2012). The sphingosine-1-phosphate transporter Spns2 expressed on endothelial cells regulates lymphocyte trafficking in mice. J Clin Invest.

[23] Nagahashi M (2013). Spns2, a transporter of phosphorylated sphingoid bases, regulates their blood and lymph levels, and the lymphatic network. Faseb J.

[24] Hisano Y, Nishi T, Kawahara A (2012). The functional roles of S1P in immunity. J Biochem.

[25] Nijnik A (2012). The role of sphingosine-1-phosphate transporter Spns2 in immune system function. J Immunol.

[26] Angers M, Uldry M, Kong D, Gimble JM, Jetten AM (2008). Mfsd2a encodes a novel major facilitator superfamily domain-containing protein highly induced in brown adipose tissue during fasting and adaptive thermogenesis. Biochem J.

[27] Huang Y, Lemieux MJ, Song J, Auer M, Wang DN (2003). Structure and mechanism of the glycerol-3-phosphate transporter from *Escherichia coli*. Science.

[28] Kawahara A (2009). The sphingolipid transporter spns2 functions in migration of zebrafish myocardial precursors. Science.

[29] Sato, K. *et al*. Critical role of ABCA1 transporter in sphingosine 1-phosphate release from astrocytes. *J Neurochem*, 10.1111/j.1471-4159.2007.04958.x (2007). 10.1111/j.1471-4159.2007.04958.x 17931360

[30] Mitra, P. *et al*. Role of ABCC1 in export of sphingosine-1-phosphate from mast cells. *Proc Natl Acad Sci USA* (2006). 10.1073/pnas.0603734103 PMC1637593 17050692

[31] Honig SM (2003). FTY720 stimulates multidrug transporter- and cysteinyl leukotriene-dependent T cell chemotaxis to lymph nodes. J Clin Invest.

[32] Takabe K (2010). Estradiol induces export of sphingosine 1-phosphate from breast cancer cells via ABCC1 and ABCG2. J Biol Chem.

[33] Nieuwenhuis B (2009). Involvement of the ABC-transporter ABCC1 and the sphingosine 1-phosphate receptor subtype S1P(3) in the cytoprotection of human fibroblasts by the glucocorticoid dexamethasone. J Mol Med (Berl).

[34] Ito S (2013). Increased plasma sphingosine-1-phosphate in obese individuals and its capacity to increase the expression of plasminogen activator inhibitor-1 in adipocytes. Coron Artery Dis.

[35] Tanfin Z, Serrano-Sanchez M, Leiber D (2011). ATP-binding cassette ABCC1 is involved in the release of sphingosine 1-phosphate from rat uterine leiomyoma ELT3 cells and late pregnant rat myometrium. Cell Signal.

[36] Lee YM, Venkataraman K, Hwang SI, Han DK, Hla T (2007). A novel method to quantify sphingosine 1-phosphate by immobilized metal affinity chromatography (IMAC). Prostaglandins Other Lipid Mediat.

[37] Vu, T. M. *et al*. Mfsd2b is essential for the sphingosine-1-phosphate export in erythrocytes and platelets. *Nature*, 10.1038/nature24053 (2017). 10.1038/nature24053 29045386

[38] Hooper M, Hardy K, Handyside A, Hunter S, Monk M (1987). HPRT-deficient (Lesch-Nyhan) mouse embryos derived from germline colonization by cultured cells. Nature.

[39] Min JK, Yoo HS, Lee EY, Lee WJ, Lee YM (2002). Simultaneous quantitative analysis of sphingoid base 1-phosphates in biological samples by o-phthalaldehyde precolumn derivatization after dephosphorylation with alkaline phosphatase. Anal Biochem.

