# Supplementary material for: MFSD2B is a sphingosine 1-phosphate transporter in erythroid cells
Source: Sci Rep. 2018 Mar 21;8:4969. doi: 10.1038/s41598-018-23300-x (PMC5862976; doi:10.1038/s41598-018-23300-x)
Supplement: Supplementary file 1 — Supplemental Figures [file 41598_2018_23300_MOESM1_ESM.pdf]

MFSD2B is a sphingosine 1-phosphate transporter in erythroid cells

Naoki Kobayashi [1,2], Shoko Kawasaki-Nishi [3], Masato Otsuka [1], Yu Hisano[3], Akihito Yamaguchi [4], and Tsuyoshi Nishi [3,5*]

[1]Department of Biochemistry, Faculty of Pharmaceutical Sciences, Setsunan University, Hirakata, Osaka 573-0101, Japan. [2]Faculty of Pharmaceutical Science, Teikyo Heisei University, Nakano, Nakano-ku, Tokyo 164-8530, Japan. [3]Department of Biomolecular Science and Regulation and [4]Department of Cell Membrane Structural Biology, Institute of Scientific and Industrial Research, Osaka University, Ibaraki, Osaka 567-0047, Japan. [5]Faculty of Pharmaceutical Science, Osaka University, Suita, Osaka 565-0871, Japan.

***Corresponding author:** Tsuyoshi Nishi, PhD. Department of Biomolecular Science and Regulation, Institute of Scientific and Industrial Research, Osaka University, Ibaraki, Osaka, Japan. Tel.: +81-6-6879-8547, Fax: +81-6-6879-8549, e-mail: tnishi@sanken.osaka-u.ac.jp

Running title: S1P transporter in erythroid cells

Abbreviations: S1P, sphingosine 1-phosphate

Supplemental Table 1.
List of tested genes that encode membrane proteins expressed in erythrocytes

Name	Aliases	GeneID	S1P export activity	Reference
Slc1a7	Eaat5	NM_146255	None	1,2
Slc2a1	Glut1	NM_006516	None	1,2
Slc2a3	Glut3	NM_006931	None	1,3
Slc4a1	Band3	NM_000342	None	1,2
Slc12a7	Kcc4	NM_006598	None	1,4
Slc14a1	Utb1	NM_015865	None	1,2
Slc16a1	Mct1	NM_003051	None	1,5
Slc19a1	RFC1	NM_031196	None	1,6
Slc22a4	OCTN1	NM_003059	None	7
Slc27a4	FATP4	NM_005094	None	1
Slc29a1	ENT1	NM_001078177	None	1,2
Slc40a1	Fpn1, MTP1	NM_014585	None	1
Slc43a1	Lat3	NM_003627	None	1
Slc43a2	Lat4	NM_152346	None	1
Abcc1	Mrp1	NM_008576	None (ref. 8)	1,9,10
Abcb6		NM_005689	None	1,11
Abcc4	Mrp4	NM_001105515	None	1,12
Abcc5	Mrp5	NM_013790	None	12,13
Abcg2	Bcrp1	XM_011242362	None (ref. 8)	1,14
Cd36	FAT	NM_001127444	None	15
RHD		NM_016124	None	1,16
PLSCR1		NM_021105	None	1
PLSCR4		NM_001128304	None	1

1. Pasini EM, *et al. Blood* **108** 791-801 (2006)
2. Rungaldier S, *et al. Biochim. Biophys. Acta* **1828**, 956-966 (2013)
3. Haber RS, *et al. Endocrinology* **132**, 2538-2543 (1993)
4. Pan D, *et al. J. Biol. Chem.* **286**, 30492-30503 (2011).
5. Wilson MC, *et al. J. Biol. Chem.* **284**, 20011-20021 (2009)
6. Thornalley PJ, *et al. Diabetologia* **50**, 2164-2170 (2007)
7. Kobayashi D, *et al. Exp. Hematol.* **32** 1156-1162 (2004)
8. Hisano Y, *et al. J. Biol. Chem.* **286**, 1758-1766 (2011)

9. Kobayashi N, *et al*. *J. Biol. Chem.* **284**, 21192-21200 (2009)
10. Flens MJ, *et al*. *Am. J. Pathol.* **148** 1237-1247 (1996)
11. Kiss K, *et al*. *PLoS One* **7** e37378 (2012)
12. Klokouzas A, *et al*. *Eur. J. Biochem*. **270** 3696-3708 (2003)
13. Jedlitschky G, *et al*. *J. Biol. Chem.* **275**, 30069-30074 (2000)
14. Zhou S, *et al*. *Blood* **105** 2571-2576 (2005)
15. van Schravendijk MR, *et al*. *Blood* **80** 2105-2114 (1992)
16. Westhoff CM, *et al*. *Genomics* **57**, 451-454 (1999).

Supplemental Figure 1

A

ProbeName	(raw) [E14tg2a]	(raw) [MEDEP−E14]	(normalised) [E14tg2a]	(normalised) [MEDEP−E14]	(flag) [E14tg2a]	(flag) [MEDEP−E14]	GeneSymbol
A_55_P1973809	4.43891	416667.53	0	16.386673	A	P	Hbb−b1
A_55_P2038540	4.48018	371297.56	0	16.207005	A	P	Hbb−b2
A_52_P534583	3.91342	95844.02	0	14.448313	A	P	Ahsp
A_52_P52618	4.14721	91789.17	0	14.302237	A	P	Csf2rb
A_55_P2108151	4.34502	68109.336	0	13.804544	A	P	Beta−s
A_51_P146753	4.42671	49417.758	0	13.314849	A	P	Csf2rb2
A_51_P391716	3.80485	24900.723	0	12.544404	A	P	Ermap
A_55_P1966194	4.10396	25806.588	0	12.486776	A	P	Plek
A_55_P1997126	3.82371	23348.537	0	12.444414	A	P	Ctse
A_55_P2091191	4.41786	26509.668	0	12.419227	A	P	Slc28a2
A_55_P2027737	4.55893	25705.982	0	12.329463	A	P	Il1rl1
A_51_P469568	3.91679	19126.05	0	12.1219225	A	P	Cldn13
A_51_P299062	3.53398	16937.402	0	12.094973	A	P	Kel
A_55_P2097478	4.12675	19026.52	0	12.039061	A	P	Gda
A_30_P01027839	8.78612	38907.273	0	11.980869	A	P	
A_55_P2091193	4.02865	14453.658	0	11.677192	A	P	
A_55_P1952618	3.50826	11790.741	0	11.582953	A	P	Ear2
A_51_P327451	3.9468	12856.033	0	11.537816	A	P	Alas2
A_52_P317653	14.5577	46790.08	0	11.518543	A	P	Car1
A_55_P2017377	3.53607	9031.465	0	11.186938	A	P	Olfr424
A_55_P2099952	9.63934	24398	0	11.173883	A	P	Car1
A_51_P474053	4.25061	10572.791	0	11.148741	A	P	Erv3
A_55_P2094925	16.1218	36748.91	0	11.022818	A	P	Srgn
A_51_P346575	3.76036	7628.1504	0	10.8545885	A	P	Rhag
A_55_P2158498	4.35388	8309.462	0	10.76658	A	P	Btk
A_55_P2153382	4.40749	7440.703	0	10.58961	A	P	Ermap
A_51_P363400	4.06791	6861.9365	0	10.588454	A	P	Prg2
A_55_P2063376	13.6956	22671.178	0	10.56127	A	P	Add2
A_51_P333274	8.5435	14012.951	0	10.547989	A	P	Gzmb
A_51_P358700	3.631	5508.8154	0	10.435502	A	P	Olfr1221
A_55_P2040026	9.91847	13579.585	0	10.287377	A	P	Itga4
A_51_P417891	3.54988	4817.0986	0	10.274521	A	P	Trim10
A_51_P237865	6.53479	8347.173	0	10.187273	A	P	Il4
A_52_P295104	7.2514	8271.145	0	10.023954	A	P	Smim5
A_51_P246543	3.92724	4006.329	0	9.862893	A	P	Ebf3
A_51_P312336	3.56686	3579.2744	0	9.83914	A	P	Slc14a1
A_51_P153995	4.31805	4272.8184	0	9.818935	A	P	Gp9
A_55_P2159746	4.35529	4038.0034	0	9.724999	A	P	Mfsd2b
A_52_P54261	3.7172	3378.8884	0	9.696459	A	P	Tmem56
A_55_P2006869	3.89467	3519.9185	0	9.688167	A	P	Fyb

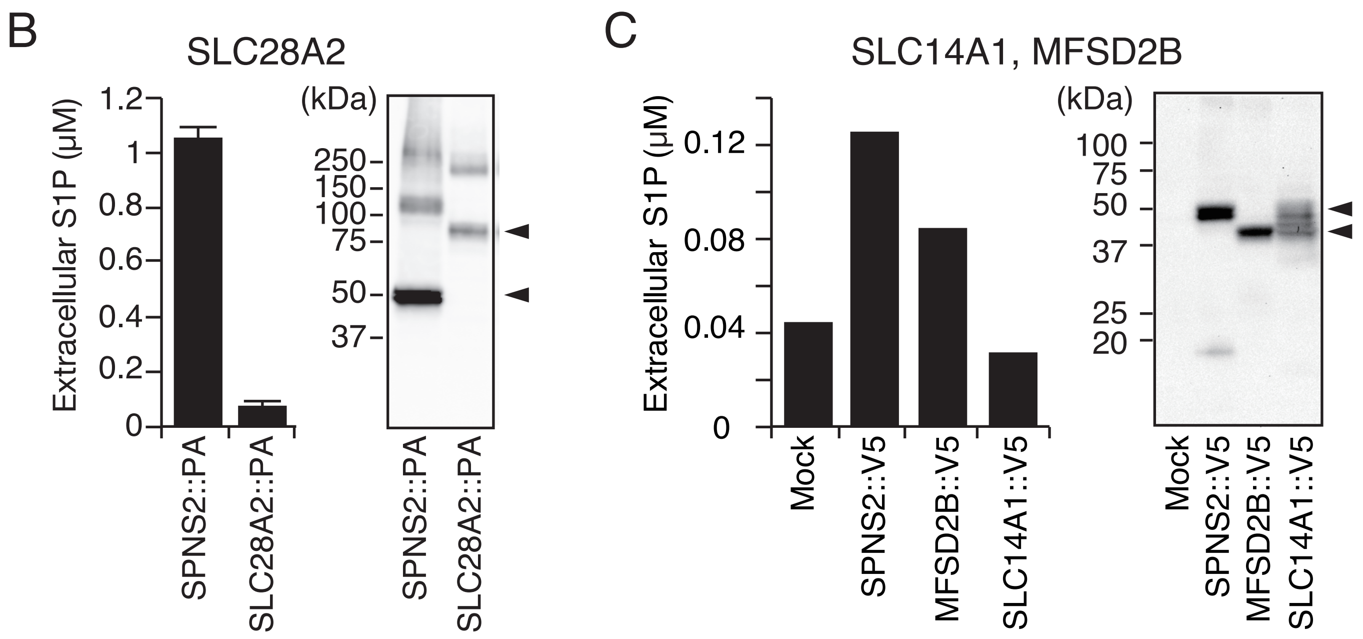

Supplemental Figure 2

```
m Mfsd2b    1                       MSVPHGPTPAPVAEPHTQEPGSDKRDGRLSVCTKVCYG    38
h Mfsd2b    1             MAAPPAPAAKGSPQPEPHAPEPGPGSAKRGREDSRAGRLSFCTKVCYG    48
m Mfsd2a    1           MAKGEGAESGSAAGLLPTSILQASERPVQVKKEP-KKKQQLSICNKLCYA    49
h Mfsd2a    1           MAKGEGAESGSAAGLLPTSILQSTERPAQVKKEPKKKKQQLSVCNKLCYA    50
                                          . .      ..   .     .  .** * * **

                TM1                                  TM2
m Mfsd2b   39 IGGVPNQVASSASAFYLQLFLLDVAQIPAAQVSLALFGGKVSGAVADPVAGFFINKSRRT    98
h Mfsd2b   49 IGGVPNQIASSATAFYLQLFLLDIAQIPAAQVSLVLFGGKVSGAAADPVAGFFINRSQRT   108
m Mfsd2a   50 VGGAPYQLTGCALGFFLQIYLLDVAKVEPLPASIILFVGRAWDAFTDPLVGFCISKSSWT   109
h Mfsd2a   51 LGGAPYQVTGCALGFFLQIYLLDVAQVGPFSASIILFVGRAWDAITDPLVGLCISKSPWT   110
               ** * *    *  * **  ***.*.      *  ** *    *  **  *. * .*  *
                                                        D93 D97

                            TM3                                      TM4
m Mfsd2b   99 GSGRLMPWALGCMPLIALAYFFLWFLPPFTSLRG-----LWYTSFYCLFQALATFFQVPY   153
h Mfsd2b  109 GSGRLMPWVLGCTPFIALAYFFLWFLPPFTSLRG-----LWYTTFYCLFQALATFFQVPY   163
m Mfsd2a  110 RLGRLMPWIIFSTPLAIIAYFLIWFVPDFPSGTESSHGFLWYLLFYCLFETLVTCFHVPY   169
h Mfsd2a  111 CLGRLMPWIIFSTPLAVIAYFLIWFVPDFPHGQT-----YWYLLFYCLFETMVTCFHVPY   165
                ******    .*.   ***  ** * * .        .**  *****  . * * ***

                                                  TM5
m Mfsd2b  154 TALTMILTPSPRERDSATAYRMTMEMAGTLMGATVHGLIVSSAHGS---------QRCED   204
h Mfsd2b  164 TALTMLLTPCPRERDSATAYRMTVEMAGTLMGATVHGLIVSGAHRP---------HRCEA   214
m Mfsd2a  170 SALTMFISTEQSERDSATAYRMTVEVLGTVIGTAIQGQIVGQAKAPCLQDQNGSVVVSEV   229
h Mfsd2a  166 SALTMFISTEQTERDSATAYRMTVEVLGTVLGTAIQGQIVGQADTPCFQDLNSSTVASQS   225
               ****       ***********.*  **  *    * **  *  .            .

                                          TM6
m Mfsd2b  205 TVHPRSPAVSPDVARLYCIAAAVVALTYPVCGSLLCLGVKEQPDTSAPASGQGLNFFTGL   264
h Mfsd2b  215 TATPGPVTVSPNAAHLYCIAAAVVVVTYPVCISLLCLGVKERPDPSAPASGPGLSFLAGL   274
m Mfsd2a  230 ANRTQSTASLKDTQNAYLLAAGIIASIYVLCAFILILGVREQRELYESQQAESMPFFQGL   289
h Mfsd2a  226 ANHTHGTTSHRETQKAYLLAAGVIVCIYIICAVILILGVREQREPYEAQQSEPIAYFRGL   285
                              *  ** .    *  *   * *** *.             .. **

                                  TM7                                TM8
m Mfsd2b  265 AITSQHPPYLSLVVSFLFISAAVQVEQSYLVLFCTHASKLQDHVQNLVLIILVSAVLSTP   324
h Mfsd2b  275 SLTTRHPPYLKLVISFLFISAAVQVEQSYLVLFCTHASQLHDHVQGLVLTVLVSAVLSTP   334
m Mfsd2a  290 RLVMGHGPYVKLIAGFLFTSLAFMLVEGNFALFCTYTLDFRNEFQNLLLAIMLSATFTIP   349
h Mfsd2a  286 RLVMSHGPYIKLITGFLFTSLAFMLVEGNFVLFCTYTLGFRNEFQNLLLAIMLSATLTIP   345
               .   * ** .*   *** * *        .****         *.* * .  ** .  *

                                     TM9                    TM10
m Mfsd2b  325 LWEWVLQRFGKKTSAF-GICVMVPFSILLAAVPS-APVAYVVAFVSGVSIAVSLLLPWSM   382
h Mfsd2b  335 LWEWVLQRFGKKTSAF-GIFAMVPFAILLAAVPT-APVAYVVAFVSGVSIAVSLLLPWSM   392
m Mfsd2a  350 IWQWFLTRFGKKTAVYIGISSAVPFLILVALMERNLIVTYVVAVAAGVSVAAAFLLPWSM   409
h Mfsd2a  346 IWQWFLTRFGKKTAVYVGISSAVPFLILVALMESNLIITYAVAVAAGISVAAAFLLPWSM   405
               * * * ******    **   *** ** *       . *.**   *.* *   ******

                                             TM11
m Mfsd2b  383 LPDVVDDFQLQHRCGPGVETIFYSSYVFFTKLSGAGALGISTLSLEFAGYEAGACQQAEE   442
h Mfsd2b  393 LPDVVDDFQLQHRHGPGLETIFYSSYVFFTKLSGACALGISTLSLEFSGYKAGVCKQAEE   452
m Mfsd2a  410 LPDVIDDFHLKHPHSPGTEPIFFSFYVFFTKFASGVSLGVSTLSLDFANYQRQGCSQPEQ   469
h Mfsd2a  406 LPDVIDDFHLKQPHFHGTEPIFFSFYVFFTKFASGVSLGISTLSLDFAGYQTRGCSQPER   465
              **** *** * . . .* * ** * ******      **.***** *..*    * * *
                                            K436

                            TM12
m Mfsd2b  443 VVVTLKVLIGAVPTCMILIGLCILLVGPTPKMPRQDTSSQLSLRRRTSYSLA           494
h Mfsd2b  453 VVVTLKVLIGAVPTCMILAGLCILMVGSTPKTPSRDASSRLSLRRRTSYSLA           504
m Mfsd2a  470 VKFTLKMLVTMAPIILILLGLLLFKLYPIDEEKRRQNKKALQALREEASSSGCSDTDSTE   529
h Mfsd2a  466 VKFTLNMLVTMAPIVLILLGLLLFKMYPIDEERRRQNKKALQALRDEASSSGCSETDSTE   525
              *  **. *    *   ** **      .     ..     *   *    *

m Mfsd2a  530 LASIL                                                          534
h Mfsd2a  526 LASIL                                                          530
```

Supplemental Figure 3

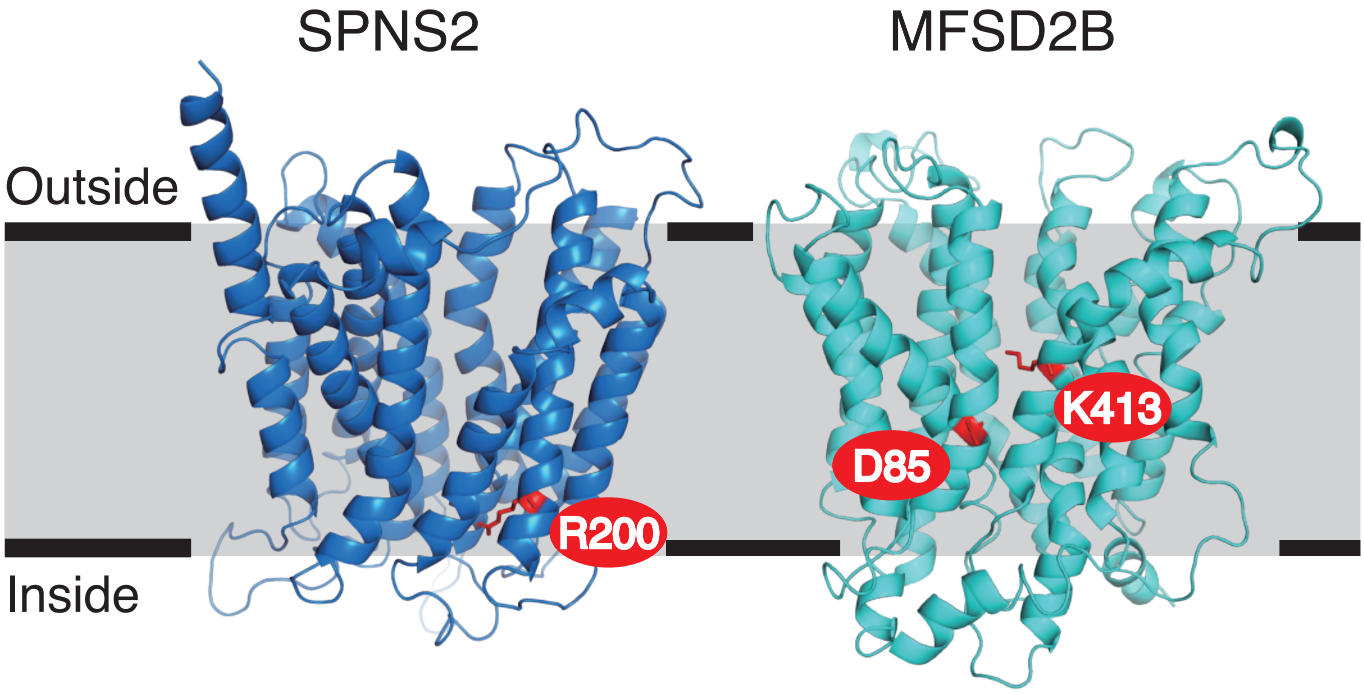

Spplemental Figure 4

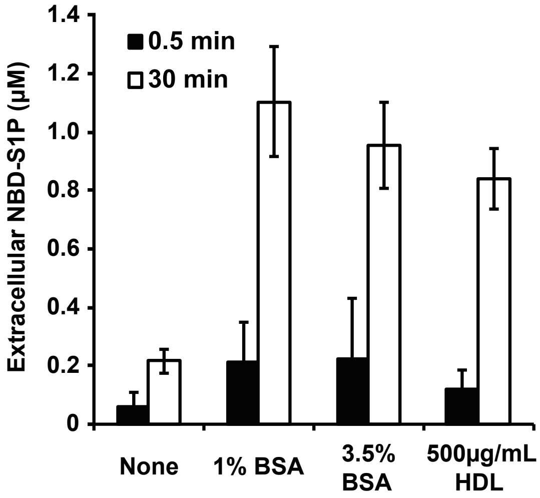

Spplemental Figure 5

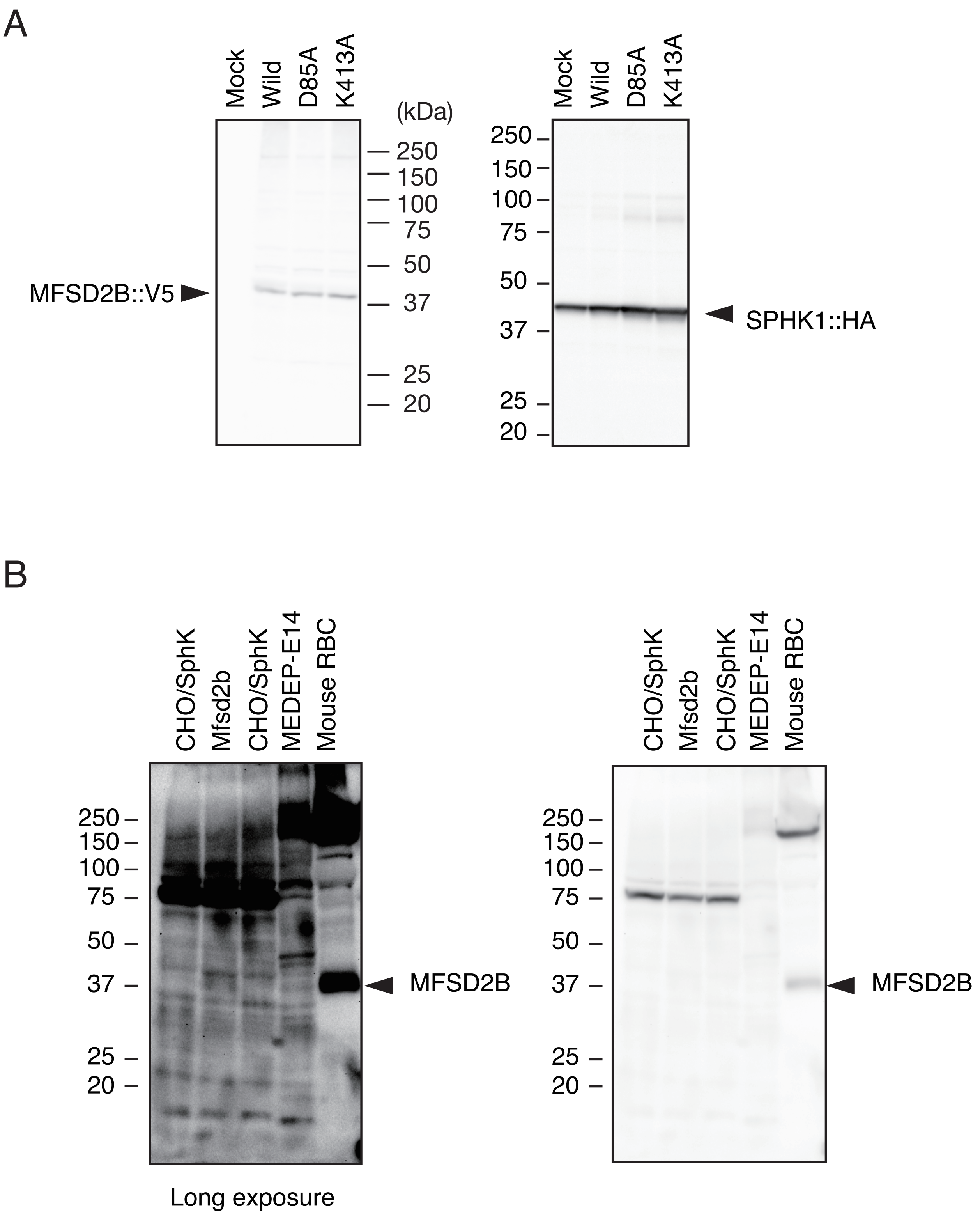

Spplemental Figure 6

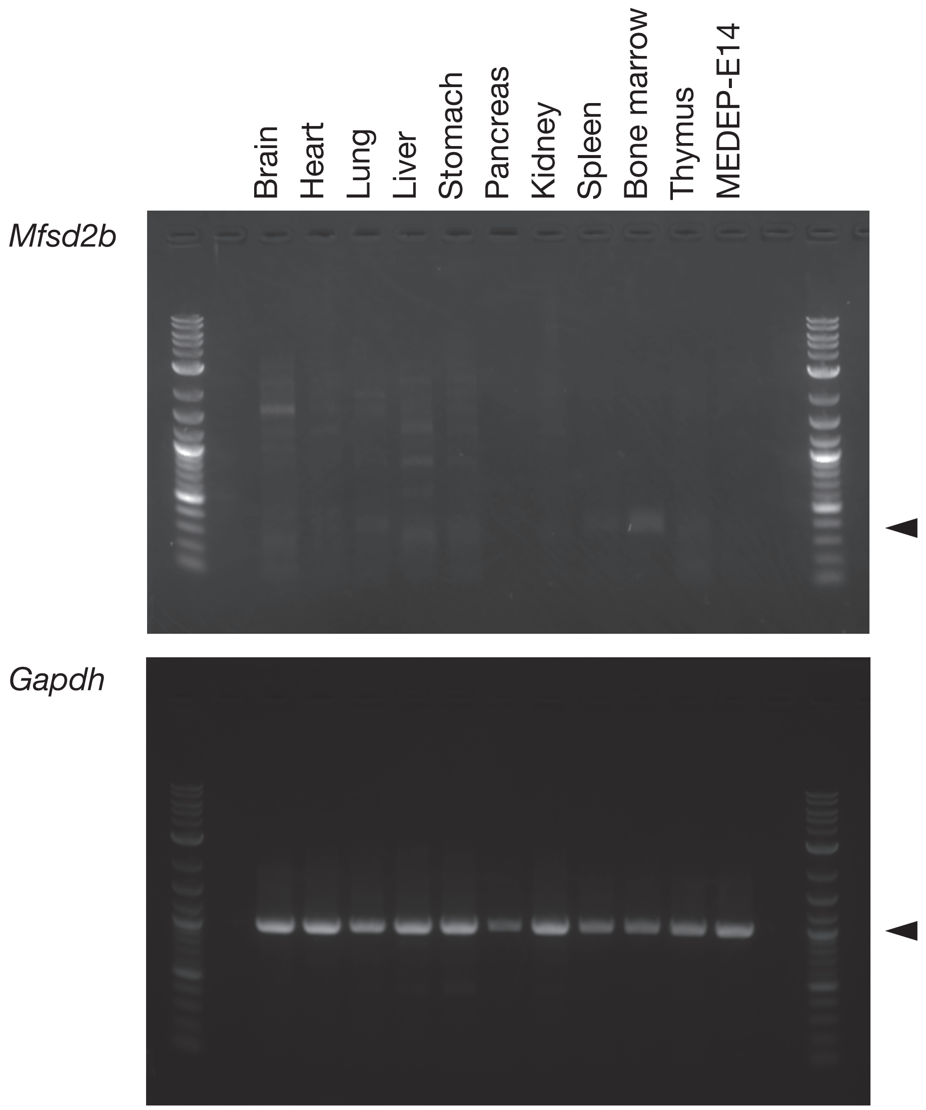

Supplemental figure legends

Supplemental Figure 1. Identification of the S1P transporter in MEDEP-E14 cells. A. From the microarray analysis data of MEDEP-E14 and E14TG2a, genes detected in MEDEP-E14 but not in E14TG2a were selected. Putative transporter genes are indicated by a yellow box. B. *Slc28a2* cDNA was cloned into the pcDNA5/FRT mammalian expression vector, transfected in CHO/SPHK1 cells and selected with hygromycin B (500 μg/ml) to isolate the cells stably expressing PA-tagged SLC28A2. V5-tagged SPNS2, SLC14A1 or MFSD2B was transiently expressed in CHO/SPHK1 cells by transfecting with mammalian expression vectors containing each gene. The expression of each protein was detected with an anti-PA tag or anti-V5 tag antibody. S1P export activity was measured in F12 releasing medium containing 5 μM sphingosine. After incubation at 37°C for two hours, the amount of S1P in the medium was quantified.

Supplemental Figure 2. Amino acid sequences of mouse and human MFSD2 proteins.
The amino acid sequences of mouse MFSD2B (mMFSD2B), human MFSD2B (hMFSD2B), mouse MFSD2A (mMFSD2A) and human MFSD2A (hMFSD2A) were aligned using ClustalW analysis. Putative trans-membrane regions of the proteins are indicated as TM1 to TM12. The mutated residues that have been reported in human MFSD2A are indicated in red. Identical residues and similar residues between the proteins are indicated with an asterisk and dot, respectively.

Supplemental Figure 3. Molecular modeling of SPNS2 and MFSD2B.
The structures of SPNS2 and MFSD2B were constructed by using the data of the crystal structures for GlpT and MelB, respectively. Amino acid residues that are important for S1P transport in SPNS2 and MFSD2B are indicated in red.

Supplemental Figure 4. BSA and HDL dependent export of NBD-S1P from MEDEP-E14 cells. MEDEP-E14 cells were pre-incubated with 5 μM NBD-sphingosine at 37°C for 30 min. Then, the cells were washed once with IMDM medium and incubated with different concentrations of BSA (1 and 3.5%) or HDL (500μg/ml). After incubation at 37°C for 30 min (30 min) or without incubation (0.5 min), the cell suspensions were centrifuged briefly. The lipids in the medium and the

cells were extracted and analyzed by TLC. The amounts of NBD-S1P in the medium and the cells were quantified.

Supplemental Figure 5. Full-length blot images of Figure 6C and Figure 8B

A. Membrane fractions from each of the transporters expressed in cells were isolated and subjected to Western blotting with anti-V5-HRP mAb. Expression of HA-tagged SphK1 was detected with anti-HA mAb labeled with HRP and was used as a loading control for each sample. B. Expression of MFSD2B protein in the mock-transfected CHO/SPHK1 cells (CHO/control), CHO/SPHK1 cells stably expressing MFSD2B (CHO/MFSD2B), MEDEP-E14 cells and mouse erythrocytes (RBC) was detected by Western blotting with rabbit anti-mouse MFSD2B polyclonal antibodies. Following removal of unbound primary antibodies by washing, blots were incubated with horseradish peroxidase-conjugated secondary antibodies (Jackson ImmunoResearch) and developed using a chemiluminescence detection method (Nakarai). Chemiluminescent images were obtained by ImageQuant LAS 500 (GE Helthcare).

Supplemental Figure 6. Full-length gel images of Figure 8A

Total RNA isolated from various mouse tissues and MEDEP-E14 cells was subjected to RT-PCR. Amplified products of *Mfsd2b* and *Gapdh* were analyzed by 1.5% agarose gel electrophoresis. Gels were stained with ethidium bromide and fluorescent images were obtained by ImageQuant LAS 500 (GE Helthcare).
